# The Immunopathogenesis of Alzheimer’s Disease Is Related to the Composition of Gut Microbiota

**DOI:** 10.3390/nu13020361

**Published:** 2021-01-25

**Authors:** Friedrich Leblhuber, Daniela Ehrlich, Kostja Steiner, Simon Geisler, Dietmar Fuchs, Lukas Lanser, Katharina Kurz

**Affiliations:** 1 Department of Gerontology, Neuromed Campus, Kepler University Clinic, Linz A-4020, Austria; friedrich.leblhuber@liwest.at (F.L.); daniela.ehrlich@kepleruniklinikum.at (D.E.); Kostja@kostja.at (K.S.); 2 Institute of Biological Chemistry, Biocenter, Medical University of Innsbruck, Innsbruck A-6020, Austria; simon.geisler@i-med.ac.at (S.G.); dietmar.fuchs@i-med.ac.at (D.F.); 3 Department of Internal Medicine, Medical University of Innsbruck, Innsbruck A-6020, Austria; lukas.lanser@i-med.ac.at

**Keywords:** Alzheimer’s disease, gut microbiome, oral pathobionts, neuroinflammation, microbial amyloid, therapeutic intervention

## Abstract

The microbiota–gut–brain axis plays an important role in the development of neurodegenerative diseases. Commensal and pathogenic enteric bacteria can influence brain and immune system function by the production of lipopolysaccharides and amyloid. Dysbiosis of the intestinal microbiome induces local and consecutively systemic immune-mediated inflammation. Proinflammatory cytokines then trigger neuroinflammation and finally neurodegeneration. Immune-mediated oxidative stress can lead to a deficiency of vitamins and essential micronutrients. Furthermore, the wrong composition of gut microbiota might impair the intake and metabolization of nutrients. In patients with Alzheimer’s disease (AD) significant alterations of the gut microbiota have been demonstrated. Standard Western diet, infections, decreased physical activity and chronic stress impact the composition and diversity of gut microbiota. A higher abundancy of “pro-inflammatory” gut microbiota goes along with enhanced systemic inflammation and neuroinflammatory processes. Thus, AD beginning in the gut is closely related to the imbalance of gut microbiota. Modulation of gut microbiota by Mediterranean diet, probiotics and curcumin can slow down cognitive decline and alter the gut microbiome significantly. A multi-domain intervention approach addressing underlying causes of AD (inflammation, infections, metabolic alterations like insulin resistance and nutrient deficiency, stress) appears very promising to reduce or even reverse cognitive decline by exerting positive effects on the gut microbiota.

## 1. Amyloid Beta and Neuroinflammation in Alzheimer’s Disease

Alzheimer’s disease (AD) is the most common form of dementia in older aged populations worldwide. An increasing incidence and prevalence of AD in Western societies will go along with a high socioeconomic burden in the next few decades, if no effective prevention strategies are developed [1,2]. Extracellular amyloid beta (Aβ) deposition (neuritic plaques) and intracellular accumulation of hyperphosphorylated tau (p-Tau; neurofibrillary tangles) still are the primary neuropathological hallmarks of AD [3]. Aβ is formed from the amyloid precursor protein (APP), a multifunctional protein that is highly conserved throughout evolution and expressed in neurons and glia. In the human hippocampus, Aβ is essential for learning and memory formation and enhances memory consolidation [4]. Thus, Aβ production is necessary for learning processes which are impaired in patients with AD. When AD progresses, memory and spatial orientation worsen continuously [5] until patients cannot care for themselves anymore and are in need for high maintenance.

Deposits of Aβ have been demonstrated in the brains of patients with AD [6], but also in the brains of elder patients without dementia [7]. Aβ can activate the microglia—the brain’s innate immune cells to induce a neuroinflammatory response. This initially beneficial response in the central nervous system (CNS) can have detrimental effects, if it continues for longer periods, as it triggers the chronic release of pro-inflammatory cytokines [8]. These cytokines then nourish chronic neuroinflammatory cascades, which over the course of years lead to irreversible neurodegeneration, especially in the hippocampus.

However, recent data indicate that not only neuro-inflammatory cascades, but also alterations of gut microbiota play a crucial role in the development of neurodegeneration: data investigating gut microbiota in humans strongly suggest that neuroinflammation is related strongly to gut dysbiosis (as reviewed in the following chapters).

## 2. Altered Gut Microbiota in Alzheimer´s Disease

Each individual, akin to fingerprints, has its own microbiome that influences his health importantly [9,10]. Mainly the intestines, further skin, lung, the oral and nasal cavity and other mucosal environments are colonized by an enormous amount of microorganisms, including bacteria, viruses, fungi and parasites [11,12,13]. Recent studies describe more than 5000 bacterial taxa residing in the human gut, mostly *Firmicutes* and *Bacteroidetes*. The number of these microorganisms may exceed 10^14^—this means that we have about 10 times more bacteria in us than human cells. The amount of genomic content of our microbiota is even 100 times higher than the one of the human genome [14]—although these figures are questioned by others [15]. 

Vaginally delivered babies mirror similar microbiota as the mother’s vagina, whereas babies delivered via cesarean section show intestinal taxa similar to those associated with the skin. At the age of around 2.5 years, the diversity of intestinal microbiota reflects those of adult microbiota and stays relatively stable during adulthood [16]. Secretory products of microbiota can be powerful pro-inflammatory and innate-immune activators [17], on the other hand a diverse and balanced gut microbiome can also produce anti-inflammatory short chain fatty acids (SCFAs) [18].

Gut microbiota comprise a community of microorganisms which have many important functions for the health of their host (see other reviews [19]): they defeat invading pathogens, metabolize nutrients, produce neurotransmitters and regulate the immune system. During recent years, the crucial role of intestinal microbiota for the development of different diseases like infections, metabolic, autoimmune and mental diseases has been recognized [20]. A reduced diversity and the wrong composition of the intestinal microbiome appear to play an important role in the pathogenesis of these diseases. 

Actually, recent studies showed an association of the development of neurodegenerative diseases like AD with alterations of the gut microbiota [19,21]. In fact, studies with AD patients and healthy controls conducted at the Alzheimer´s disease Research Center showed significant differences regarding the composition of the intestinal microbiome [22]. Patients with AD had decreased numbers of *Firmicutes* and *Actinobacteria*, while the number of bacteria belonging to the *Proteobacteria* and *Bacterioidetes phyla* were increased and linked with the severity of AD [22]. Furthermore, *Enterobacteriaceae* were associated with the presence and progression of AD. The number of “pro-inflammatory bacteria” like *Gammaproteobacteria*, *Enterobacteriales* and *Enterobacteriaceae* of phylum *Proteobacteria* increased steadily from healthy controls to mild cognitive impairment and dementia stage. These changes also correlated significantly with the clinical severity of AD [23].

Interestingly, the levels of various intestinal bacteria were correlated with cerebrospinal markers for AD like Ab42/Ab40 or p-Tau [22]. Furthermore, a Chinese study confirmed significant differences in the composition of gut microbiota between healthy individuals and AD patients [24]. However, the qualitative changes were not comparable in China and the USA. Probably, various differences regarding lifestyle, dietary habits, ethnicity or comorbidities explain this finding best. 

Furthermore, data from animal experiments strongly suggest that microbiota are involved in AD development: preclinical studies in germ-free mice showed a significant reduction of cerebral Aβ when compared to mice with intestinal microbiota [25]. Moreover, transplantation of fecal samples from AD mice to germ-free mice was associated with a significant increase of Aβ levels [26].

## 3. Gut Dysbiosis, Immune System and Neuroinflammation in Alzheimer’s Disease

The immune system is modulated by the gut microbiota: more than 70% of the available immune cells are present in the gut [27] and dysbiosis of the gut microbiota influences the “human ecosystem” dramatically: as bacteria regulate the permeability of the intestines, e.g., by secreting mucus, overgrowth of “the wrong” microorganisms or just reduced diversity of the microbiota can promote a “leaky gut”. In that case invading pathogens, lipopolysaccharides (LPS) and also certain metabolites can leave the gut and enter the bloodstream. In response to this microbial “attack” the immune system releases pro-inflammatory cytokines to inhibit bacteremia.

**Figure 1:**
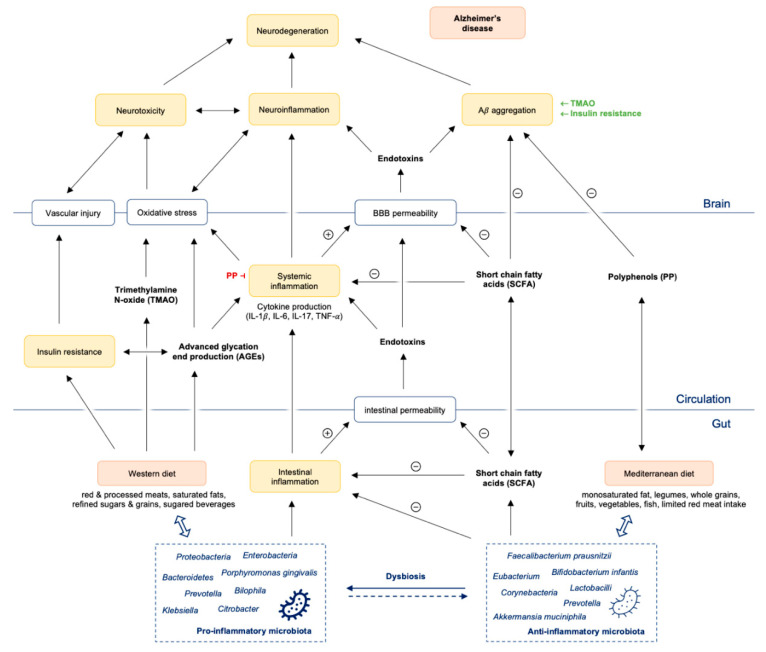
Gut dysbiosis promotes intestinal and systemic inflammation with consequently Aβ aggregation and neuroinflammation finally leading to neurodegeneration and Alzheimer’s disease. Abbreviations: Aβ = amyloid beta; PP = polyphenols; SCFA = short chain fatty acids; TMAO = trimethylamine *N*-oxide; IL = interleukin; TNF = tumor necrosis factor; BBB = blood brain barrier.

While this strategy of the immune system is necessary and very effective in acute situations, chronic (persistent) inflammatory reactions of the gut mucosa can overcharge the immune system and lead to a chronic, over-active, but ineffective immune response. They are supposed to go along with the development of many chronic diseases, like inflammatory bowel disease, diabetes, atherosclerosis, and also neurodegenerative diseases [19]. Chronic microbial translocation can also drive distant organ failure and injury [28]. Furthermore, bacterial translocation and secretion of pro-inflammatory cytokines significantly impair the integrity of the blood–brain barrier (BBB) and induce neuroinflammatory cascades [29,30,31]. Microbes from the periphery can get into the brain or periodically reactivate microbes from latent brain tissue infection, thus switching to detrimental chronic inflammation with Aβ overproduction and deposition as senile plaques. Both, microbes and their products amyloid and LPS [32] can infiltrate into the brain and initiate neuroinflammation. Dysbiosis of the gut microbiota therefore does not only affect the intestines, e.g., by a decreased formation of SCFA [33,34], and the production of vitamins [35], but also influences the balance between microbiota and immune cells crucially (Figure 1).

Increasing evidence suggests that neuroinflammation with its immune components may be a central cause of Aβ aggregation, tau hyperphosphorylation and finally neuronal death [36,37]. Pro-inflammatory cytokines—initially beneficial and neuroprotective—finally cause progressive neurodegeneration [38]. Thus, inflammatory reactions could be both beneficial, called euflammation [39] as well as detrimental to the brain: depending on the extent and also stage of activation neurodegeneration can be enhanced mildly or strongly.

Chronic inflammatory reactions result in microglial overactivation by releasing large amounts of inflammatory agents as seen in different neuropathologies [40]. Finally, the imbalance of these inflammatory responses initiates the neurodegenerative process [41,42]. Consecutive neuroinflammatory responses can then further enhance neurodegenerative processes [36,43]. In conditions of dysbiosis, triggering receptors expressed on myeloid cells (TREM-1/2) were described, bridging inflammatory processes in intestinal and neurodegenerative disorders by spreading inflammatory compounds through the microbiota–gut–brain axis [44]. Thus, in addition to the primary risk factors for AD like advancing age [45], chronic intestinal inflammation may play an important role as well [44,46].

Intestinal inflammation can be measured by assessing stool calprotectin concentration, which is found increased in demented patients [47]. Calprotectin levels are also significantly increased in the cerebrospinal fluid and the brain of AD patients, promoting amyloid aggregation and co-aggregation with Aβ [37,48]. Signs of immune activation can be concurrently detected in serum (neopterin) and fecal specimens (S100A12) of AD patients [49,50]. 

Earlier studies showed that S100 calcium-binding protein A8 (S100A8) and S100A9 are co-expressed in a variety of inflammatory conditions, and elevated plasma levels of S100A9 are associated with inflammatory disorders [51]. Pro-inflammatory S100A9 is secreted by macrophages and microglia during amyloid plaque formation [48]. 

Gut dysbiosis and intestinal inflammation is directly associated with gut barrier dysfunction and increased intestinal permeability (“leaky gut”) and may therefore contribute to the process of neurodegeneration [52,53]. Dysbiosis of intestinal bacteria reduces the integrity of the intestinal barrier, thus enabling the eased invasion of pathogenic bacteria. Infection with the conditional pathogen *Citrobacter,* e.g., was shown to induce memory disorders in mice, which went along with decreased expression of brain-derived neurotrophic factor (BDNF) [54]. Interestingly, decreased BDNF levels were also demonstrated in the brain and sera of AD patients [55]. 

## 4. Influence of Microbial Metabolites on Gut Permeability, Immune System and Neurotransmitter Production in Alzheimer’s Disease

Diverse microbes of the human microbiota generate large amounts of amyloid and its bioavailability to the CNS increases with age [56]. Gram negative bacteria like *E. coli* and *Salmonella spp*. produce extracellular amyloid fibers known as curli via a multi-component secretion system [57]. Curli are potent inducers of the host inflammatory response [58]. Additionally, fungi produce LPS, amyloids and other microbial exudates [56,57]. Gut microbiota continuously produce and release extracellular protein fibers to create and maintain a biofilm, since these fibers offer protection from environmental stressors; additionally they mediate adherence to both biotic and abiotic surfaces as well [59]. 

Microbial and cerebral amyloids, biologically and chemically similar concerning pathogen-associated molecular pattern (PAMP), do not share amino acid sequences with human Aβ1–42 [56]. Anyway, they are recognized by the same receptor system, and are also activating pro-inflammatory cytokines like interleukin (IL)-17 and IL-22. This may be deleterious during aging, when the gastrointestinal mucosa, as well as the BBB become continuously more permeable [56,60,61]. These powerful microbiome-derived pro-inflammatory activators induce pro-inflammatory cytokines, complement activation, and alter immunogenicity in the brain enhancing amyloid aggregation and inflammatory reactions. Both, amyloid proteins and LPS, are strong activators of the receptor for advanced glycation end-products (RAGE) and Toll like receptors (TRLs), perpetuating chronic inflammation in AD [62]. 

However, gut microbiota do not only secrete pro-inflammatory metabolites, but they are also capable to produce diverse “anti-inflammatory” and immunomodulatory compounds like SCFA butyrate depending on their composition and diversity [63]. Commensal gut microbes are fermenting dietary fibers into SCFAs, thereby increasing the production of tight junction proteins claudin-5 and occludin, which are important for a tight and selective barrier function. In line with these data, a higher abundance of butyrate-producing bacteria and higher butyrate concentrations could be demonstrated to be protective against *Citrobacter rodentium* invasion in rodents [64]. Accordingly, supplementation with butyrate strongly inhibited the growth of *Citrobacter rodentium* [64]. These data therefore imply that butyrate can support the barrier function of intestinal epithelial cells and increase junctional integrity [65], thus reducing the invasion of enteropathogens to the bloodstream and minimizing LPS translocation in the intestines [66]. Furthermore, butyrate is able to inhibit pathways important in the production of proinflammatory cytokines [67], and also improves insulin signaling [68]. Very interestingly, a recent clinical study could show that brain amyloidosis (quantified by Florbetapir amyloid PET) was significantly associated with elevated levels of blood LPS and pro-inflammatory cytokines, and inversely correlated with the anti-inflammatory cytokine IL-10 and the SCFA butyrate [69]. Furthermore, markers of endothelial dysfunction were higher in patients with increased levels of proinflammatory cytokines, while butyrate and IL-10 levels were reduced [69]. Systemic inflammation induced by LPS might therefore enforce endothelial activation and Aβ-formation [69]. 

In obese patients, numbers of the butyrate producing bacteria called *Faecalibacterium prausnitzii* were described to be significantly lower compared to healthy normal weight individuals [70]. On the other hand, higher numbers of *Faecalibacterium prausnitzii* go along with lower concentrations of pro-inflammatory cytokines [71] and CRP [72]. Interestingly, patients with an increased BMI (>25) with higher CRP levels also had a lower abundance of *Lactobacilli* and *Bifidobacteria*, while *Escherichia coli* and *Bacteroides* species were more abundant in the same patients [73]. Obesity and insulin resistance are in fact a major risk factor for cognitive decline [74], especially in aging people. 

In aging individuals a marked reduction of microbial biodiversity is seen, which is characterized by a higher abundance of *Proteobacteria* and a decreased number of *Bifidobacteria* species, and consecutively by significantly lowered SCFA formation [41,75,76]. 

In case of age or diet-related dysbiosis influences of “beneficial gut microbial metabolites” on intestinal integrity and on the BBB get lost [29,77]. Dysbiosis therefore goes along with an increased permeability of the intestinal barriers [78]. Additionally, it also decreases the permeability of the BBB [79] and the synthesis and secretion of neurotrophic factors, such as brain derived nerve growth factor (BDNF), and *N*-methyl D-Aspartate (NMDA) receptors [80]. Reduced BDNF levels and NMDA signaling are associated with cognitive decline [80]. 

In healthy individuals, commensal microbiota furthermore produce precursors of a number of other important neurotransmitters: acetylcholine, catecholamines, gamma-aminobutyric acid, as well as tryptophan metabolites kynurenine, melatonin, and serotonin [41]. In fact, many of these neurotransmitters are centrally involved in the control of gut–brain axis signaling [81,82]. A recent review by Bosi and coworkers [83] describes how metabolites of the kynurenine pathway, e.g., quinolinic acid and kynurenic acid, influence neuronal activity in the CNS and in the periphery by exerting neurotoxic and neuroprotective effects: host kynurenine production may be influenced by the microbiota by modulation of glucocorticoid-induced TDO-activity [84] and immune-mediated IDO activity [85]. Very interestingly, in germ-free (GF) rodents, tryptophan plasma levels increase while the kynurenine-to-tryptophan ratio increases (due to IDO and TDO activity changes) [33,86,87,88]. Interestingly, administration of probiotics such as *Bifidobacterium infantis* normalizes the kynurenine-to-tryptophan ratio [87]. Dysregulation of tryptophan/kynurenine metabolism thus might contribute significantly to microbiota–gut–brain axis disorders and facilitate the development of neuropsychiatric symptoms [33]. Microbial benefits of other tryptophan catabolites were also reviewed recently [89]: indoles and tryptamine, e.g., were described by Roager and coworkers to activate the immune system, enhance the intestinal epithelial barrier and stimulate gastrointestinal motility [89]. Furthermore, these gut bacteria derived tryptophan catabolites are involved in the secretion of gut hormones, and can have systemic anti-inflammatory, anti-oxidative or toxic effects [89]. 

In AD patients increased kynurenine/tryptophan ratios going along with elevated zonulin levels (as a marker for decreased intestinal barrier function) have been shown, and supplementation with a multi-strain probiotic was effective to decrease levels of zonulin, increase the abundance of *Faecalibacterium prausnitzii* and alter the kynurenine/tryptophan ratio [90]. Probiotics might therefore exert beneficial effects on the host by modulating host tryptophan metabolism, by enhancing neurotransmitter production and probably also by supporting the proliferation of “anti-inflammatory” bacterial strains like *Faecalibacterium prausnitzii*. 

On the other hand, disbalance of the human “gut microbiota ecosystem” can also have significant effects on the enteric nervous system (ENS), a very important part of the autonomic nervous system. The ENS, which can be regarded as a “second brain”, is important for gastrointestinal (GI) motility as well as for the modulation of the intestinal immune response in health and disease. It is important for T cell activation and response to cellular danger by secreting cytokines and immunomodulatory molecules, creating a neuroinflammatory response and neuronal death mechanisms. These responses primarily are located in the ENS, but via the gut–brain–axis they can also affect the CNS [91,92]. Both in the ENS and CNS, the same misfolded proteins initiate neurodegeneration [93]. 

Additionally, recent studies demonstrate that gut microbiota impact on the neuroinflammatory inhibitory reflex mediated by the cholinergic nervous system [94]. Actually, loss of cholinergic neurons located in the nucleus basalis of Meynert is associated with cognitive deficits in AD patients [95]. 

## 5. The Role of Periodontal Infection, Other Infectious Pathogens and Antibiotics and AD

**Figure 2:**
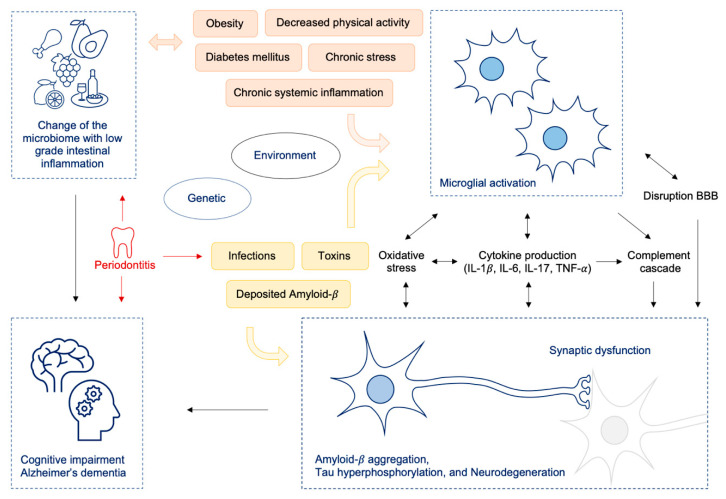
Change of the microbiome (e.g., by Western diet) resulting in intestinal dysbiosis leads to low grade inflammation in the gut and to increased intestinal and BBB permeability and consecutively to neuroinflammation and cognitive decline; oral pathobionts like *P. gingivalis* lead to oralisation of gut microbiota on the one hand, thus additionally driving gut inflammation and on the other hand promoting neuroinflammatory processes by translocation of bacteria to the brain via toxic proteases. Abbreviations: IL = interleukin, TNF = tumor necrosis factor; BBB = blood brain barrier.

Furthermore, chronic periodontal inflammation can contribute to changes in the intestinal microflora [96,97,98,99] and exacerbate host’s systemic inflammatory response [97,100,101]. In the healthy oral cavity commensals remain in physiological balance, but disturbances of this ecological state may lead to dysbiotic periodontal microbial communities and periodontitis [96]. Periodontitis is linked to several systemic diseases like atherosclerosis, diabetes mellitus and obesity [96,102,103]. The swallowed saliva of patients with periodontitis contains great amounts of bacteria: up to 10^12^ bacteria per day [98]. Periodontitis can cause secretion of bacterial pathogens like LPS, flagellin, peptidoglycan and other proinflammatory molecules, which are all modifiable risk factors for AD [104,105]. Consequently, periodontitis is a dysbiotic inflammatory disease which can mediate inflammation at local as well as distant sites including the brain [96,103,106]. Neuroinflammatory effects can be elicited via cellular, humoral and neural pathways, which all can contribute to AD pathogenesis [96,103,106] (see also Figure 2). 

In an animal study, 5 weeks of administration of *Porphyromonas gingivalis (P. gingivalis)* resulted in changes of the gut microbiota composition by increasing the intestinal Th17 cell proportions in mesenteric lymphocytes [107]. Further, *P. gingivalis* decreased insulin sensitivity and increased pro-inflammatory cytokines TNF-*α* and IL-6, causing an inflammatory response in the liver by lipid droplet formation indicating that periodontitis may affect nonalcoholic fatty liver disease (NAFLD; [108]). In a recent investigative study, a significant association between the salivary presence of *P. gingivalis* and worse results in cognitive tests was observed [101].

Infection with *Aggregatibacter actinomycetemcomitans (A. actinomycetemcomitans)*, another periodontopathogen enhanced liver steatosis in mouse models. *A. actinomycetemcomitans* antibodies also appeared higher in NAFLD patients [109]. Earlier studies showed that poor dental status and periodontal disease are linked to reduced cognitive function and AD [101,110,111]. Poole and colleagues confirmed LPS from *P. gingivalis* can enter the AD brain, suggesting an inflammatory role in AD pathology. Well in line with these data active invasion of the oral pathogen *P. gingivalis* into the brain could be demonstrated in mouse models [112,113]. Recent findings describe that oral bacteria like antibiotic resistent *Klebsiella* species can colonize the gut and induce chronic intestinal inflammation via activation of the intestinal immune system [97,99]. Thus, the oral and the gastrointestinal tract represent the magnitude of the human microbial load offering new and advancing diagnostic and therapeutic opportunities in terms of neuroinflammation and neurodegeneration [37,98,114].

However, not only bacterial translocation might contribute to neuroinflammation, but also viral infections might play a role. Viral infections often lead to a strong induction of the immune response, and elevated neopterin concentrations have been demonstrated in patients with Epstein–Barr virus (EBV) and cytomegalovirus (CMV) infections [115,116]. A recent review states that infections by herpesviruses induce Aβ production, phosphorylation of tau, oxidative stress, and neuroinflammation in general [117]. Interestingly, Aβ peptide has antimicrobial properties and is able, e.g., to inhibit HSV-1 replication [117]. Thus, reactivation of herpesviruses might initiate amyloid cascade reactions in elder vulnerable individuals, induce oxidative stress and finally neuroinflammation with the accumulation of Aβ and p-Tau [118]. Furthermore, CMV, another member of the herpes virus family, was described to promote dementia [119]. Furthermore, also an interaction between CMV and HIV-1 was described to be associated with a higher risk to develop AD [45,120,121]. As viral infections are known to induce the release of pro-inflammatory cytokines, they might also contribute to neuroinflammation by triggering systemic, but also local cytokine secretion.

The hypothesis that infectious agents trigger the development of AD was already proposed by Aloysius Alzheimer and again proposed a few years ago [106]. A recent study discusses this hypothesis again, and states that autopsy and association studies provide evidence of infectious burden (mainly bacteria like *Chlamydia pneumoniae* and *spirochetes* and herpesviruses) [122]. In line with the “infection” hypothesis 1194 patients who survived severe sepsis had substantial and persistent new cognitive impairment and functional disability [123]. The accumulation of misfolded Aβ in the brain in response to infection was proposed to be responsible for the cognitive decline of patients. On the other hand, in vitro and in vivo models experiments rather suggest that Aβ, which is also an antimicrobial agent, might be produced to prevent brain infection [124]. In fact, initially protective mechanisms to inhibit pathogen proliferation (e.g., by Aβ formation or immune activation), might lead over the course of time and during prolonged states of nutrient deficiency to excessive Aβ accumulation in the brain and chronic exhaustion of the immune system (similar to chronic immunodeficiency in HIV-infection).

As infections appear to contribute to gut dysbiosis, systemic inflammation and neuroinflammation, anti-infective treatment might be a reasonable therapeutic option. Antibiotics have saved thousands of lives, as they are very potent drugs that inhibit the proliferation of pathogens. On the other hand, they were also shown to affect cognition and memory function in rodent models and humans depending on the type of antibiotic and its targeting microbiome. Possible effects of antibiotics on the gut–brain axis and their possible influence on AD have been reviewed recently [125]: while amoxicillin, rifampicin, minocycline, rapamycin or doxycycline improved cognition, reduced Aβ deposition and accumulation of hyperphosphorylated tau, streptozotocin, ampicillin and cefepime were associated with memory deficits, reduced consciousness and confusion. [125]. In this review, a very important issue was also raised: the antibiotic streptozotocin is used to induce sporadic AD forms in animal models with effects on learning and memory performances [126]. Furthermore, streptozotocin is also employed to induce diabetes mellitus in animals [127], which is a frequent comorbidity of AD going along with early cognitive decline [128]. However, whether effects of antibiotics on cognition are due to microbiotic changes following antibiotic therapy or are caused by the antibiotic itself is not proven yet.

The use of antibiotics has furthermore also been related to depression and anxiety—which are frequently encountered in AD patients—by changing the gut microbiota and the brain–gut axis (see review by Hao et al. [129], Desbonnet et al. [130]), suggesting that we should use antibiotics wisely and only when indicated. Furthermore, long term effects of antibiotic treatment should be taken into consideration, especially in young children:

The infectiologist and microbiome researcher Martin Blaser was one of the first researchers proposing that losses of particular bacterial species of our ancestral microbiota (by over-use of antibiotics) have altered the context, in which immunological, metabolic and cognitive development occur in early life [131]. He also corroborated the hypothesis that “missing/disappearing microbes” contribute importantly to the epidemics of chronic disease [131], as the early use of antibiotics in infants goes along with substantial alterations of the gut microbiota and with the rise of the “modern plagues”: obesity, childhood diabetes, allergic diseases, chronic intestinal bowel disease and eczema [132]. 

Additionally, antibiotic treatment of very young mice induced cognitive deficits, altered dynamics of the tryptophan metabolic pathway, and significantly reduced BDNF, oxytocin and vasopressin expression in the adult brain [130]. Further studies investigating effects of antibiotics on the microbiota and cognitive and mental development of babies and infants should therefore be conducted to address this important topic.

## 6. Western Diet: Effects on Gut Microbiota, Inflammation, Metabolism and the Brain

Typically, human microbiota remain stable during longer periods of time [98,133], but changes in diet as well as antibiotic treatment can result in dysbiosis [134,135]. Standard Western diet (WD) with its high intake of refined sugars/grains, red and processed meat, saturated fats and a high consumption of sugared beverages goes along with substantial and fundamental changes of the gut microbiota (see also reviews by Noble et al. and Al Bander et al. [19,21]). The high contents of animal protein in WD appears to provide an optimal environment for anaerobic microorganisms and specific genera including *Bacteroides* and *Bilophila* [136]. On the other hand, *Prevotella* species appear to dominate in individuals eating many plants [66] with a greater diversity of the fecal microbiota compared with individuals consuming habitual WD [137]. In animal studies, feeding rodents a diet with high saturated fat, salt, and sugar consumption (“Western diet”) increased abdominal fat, insulin resistance, atherosclerosis, and inflammation [138,139,140,141]. In the following, specific effects of WD on inflammation, as well as glucose and fat metabolism will be described:

### 6.1. WD and Inflammation 

WD has a significant pro-inflammatory potential (Figure 1), which has been associated with diseases like malignant and cardiovascular disease [142,143]. A very recent meta-analysis with prospectively collected nutritional data of >200,000 people could show that a “pro-inflammatory” diet containing high amounts of refined grains/sugar/sugared beverages, processed/red meat and saturated fats goes along with higher inflammation markers like hsCRP and TNF-α Receptor 2 and a significantly worse survival [144]. Individuals consuming lots of vegetables, fruit, whole grain, green tea, wine and coffee on the other hand presented with lower inflammation markers and had a significantly lower risk for cardiovascular events [144]. Vegetables and whole grains are rich in fibers, microbiota accessible (i.e., complex) carbohydrates and potent bioactives substances which have all been demonstrated to have beneficial effects (see review by Moszak et al. [145]). In animal studies, WD also diminishes brain levels of neurotrophins such as BDNF and reduces the capability to learn [146]. 

WD may increase neuroinflammation by higher LPS production and by reducing the numbers of “anti-inflammatory” commensal bacteria [147]: mice receiving western diet fecal transplants had elevated levels of endotoxin and neuroinflammatory markers and an impaired cognitive function [147]. WD furthermore reduced the abundance of *A. muciniphilia*, an “anti-inflammatory” gut microbiota species [147].

### 6.2. WD and Its Effects on Metabolism and the Brain

The intake of too much refined grain/sugar and sugared beverages also goes along with intermittent hyperglycaemic episodes and the increased formation of advanced glycation end products (AGEs) which have also been described to play a prominent role in the development of AD [148,149]. AGEs trigger the formation of several proinflammatory cytokines like tumor necrosis factor-alpha (TNF-α), interleukin (IL)-1 and IL-6 [150], which are well established to worsen neuroinflammation and neurodegeneration processes and further decrease productive adult hippocampal neurogenesis [151]. AGEs also activate microglia by interacting with its receptor RAGE and the release of reactive oxygen species (ROS) [152] (Figure 1).

High amounts of refined sugar in the WD and constantly elevated glucose levels can lead to a disbalance of insulin signaling [153], namely insulin resistance—which might be crucial for the progression of AD, as insulin modifies neuronal activity [154] and improves memory [155]. Interestingly, insulin has also been shown to reduce neuroinflammation, e.g., by attenuating LPS-induced cytokine release in the brain and systemic inflammation [156]. Several studies have confirmed that insulin signaling is impaired in the brains of AD patients [157,158]. Cerebral glucose utilization and blood flow are significantly diminished already in the early stages of AD, in the later stages metabolic and physiological abnormalities worsen—with 55–65% reductions of cerebral blood flow [159]. Thus, altered glucose brain metabolism (similar to changes found in type 2 diabetes) occurs soon after the onset of dementia-related symptoms [160]. Several studies and epidemiological evidence have linked peripheral insulin resistance in healthy subjects and diabetes patients, respectively, with impaired cognitive performance [161,162,163]. Insulin resistance is proposed to exacerbate Aβ accumulation, tau hyperphosphorylation and disbalanced energy metabolism. Furthermore, hippocampal function is altered and inflammation is enhanced (see review by de La Monte [164]). The finding that microbiota transfer from healthy lean donors to patients with insulin resistance improves the insulin sensitivity of the recipients and the number of butyrate producing bacteria indicates that gut microbiota are involved crucially in insulin signaling [165] and the development of obesity. Furthermore, animal studies, in which the stool of obese or lean human twin pairs was transferred to mice, showed similar results: microbiome transfer from the obese twin resulted in an impaired glucose metabolism in the recipient mice [166]. In the same study, also the effects of co-housing of mice transplanted with obese and lean fecal microbiota were investigated, which showed that invasion of specific members of *Bacteroidetes* from the lean microbiota into obese microbiota occurred and was diet dependent [166].

Experiments with mice fed a high fat diet showed that *Lactobacillus intestinalis* was the only species whose abundance was negatively correlated with changes in body weight and fat mass [167]. The high fat diet favored bacterial species producing the SCFAs propionate/acetate, whose abundance was strongly correlated with adiposity [167]. High levels of the SCFAs acetate and valerate were shown recently to be associated with high levels of blood LPS and pro-inflammatory cytokines as well as an elevated amyloid uptake in patients with dementia [69]. Interestingly, high fat diets can also impair adult neurogenesis (see review by Zainuddin and Thuret [168]) and may also negatively affect the brain by altering adiponectin formation: low serum adiponectin levels were shown to be related to worse cognitive function and more progressed AD [169].

Adiponectin produced by adipose tissue does not only increase glucose uptake by adipocytes and monocytes, but also has effects on insulin sensitivity and reduces hepatic glucogenesis [169]. Furthermore, the oxidation of free fatty acids in muscles is influenced crucially by adiponectin, which also prevents an increase of serum free fatty acids and triglycerides and has anti-inflammatory and anti-atherogenic effects [169].

## 7. Mediterranean Diet: Effects on Gut Microbiota

However, diet can also exert positive effects on microbiota composition and inflammation [170]. A Mediterranean diet (MD) with high fruit and vegetable consumption, moderate consumption of poultry, fish, eggs, and dairy, and low consumption of red meat and processed foods, appears to protect against chronic inflammation and related diseases and is effective to decrease the risk for cardiovascular events [171]. 

The “PREDIMED” study [170], a prospective study investigating effects of a MD regarding cardiovascular outcome, showed that increased consumption of plant-based nutrients, such as vegetables and polysaccharides, coincided with higher counts of *Bifidobacteria* and higher total SCFA concentrations. Adherence to the MD was reflected by significantly higher levels of total SCFA. A higher ratio of *Firmicutes–Bacteroidetes* on the other hand was related to a lower adherence to the MD, while a higher abundance of *Bacteroidetes* was associated with lower animal protein intake. A high consumption of animal protein, saturated fats, and sugars (which is characteristic for a standard WD) affected gut microbiota diversity negatively. In normal-weight individuals, *Christensenellaceae* species were found more frequently compared to overweight participants. The authors concluded that diet and specific dietary components could affect microbiota composition, diversity, and activity (see also Figure 1), which may also affect host metabolism, e.g., by increasing the risk of Western diseases [170].

Adherence to the MD has been proposed as one of the top five modifiable factors against AD and cognitive decline, as several studies and also a meta-analysis have shown that high adherence to the MD is associated with a slower rate of cognitive decline [172,173,174,175]: compared with a low-fat diet, a dietary intervention with MD enhanced with either extra virgin olive oil or nuts appeared to improve cognition in middle-aged adults [176]. Furthermore, Mini Mental State examination (MMSE) and clock drawing test scores were higher for participants with MD versus a low-fat diet [176]. Therefore, two recent reviews also conclude that there are sufficient data to recommend the Mediterranean and Mediterranean-DASH (dietary approach to stop hypertension) Intervention for Neurodegenerative Delay (MIND) diets to delay the onset of AD [175,177].

In fact, recent data suggest that effects of MD are mainly due to positive effects on the gut microbiota: in the NU-AGE project dietary intervention with 1-year MD was effective to alter the gut microbiota, reduce frailty and improve cognitive function [178]. Data of 612 non-frail subjects across five European countries indicated that adherence to the MD diet provides a good ambience for bacteria, that produce butyrate, while gut microbiota that produce secondary bile acids, p-cresols, ethanol and carbon dioxide are rather diminished. Interestingly, the “beneficial, diet positive” gut microbiota taxa (*Faecalibacterium prausnitzii, Roseburia (R. hominis), Eubacterium (E. rectale, E. eligens, E. xylanophilum), Bacteroides thetaiotaomicron, Prevotella copri and Anaerostipes hadrus*) were positively associated with several markers of lower frailty and improved cognitive function, and negatively associated with inflammatory markers including CRP and IL-17 [178]. Increasing adherence to the MD increased the diversity of microbiota after one year of intervention significantly, there was a clear association between the microbiome and adherence to the MD. These changes in the intervention group were primarily driven by an increase in the intake of fibers, vitamins (C, B6, B9, thiamine) and minerals (Cu, K, Fe, Mn, Mg), while changes in the controls were associated with an increase in fat intake (saturated fats and mono-unsaturated fatty acids) relative to the MD intervention group. Interestingly, results of this study also demonstrated that there are country-specific patterns in dietary habits and that they are reflected by microbiome profiles. However, diet-responsive taxa identified across the entire cohort were largely shared across the different nationalities (Italian, UK/France, Netherlands/Poland) [178]. 

Not only the composition of nutrition, but also the quantity and the time period of nutrient intake appear to play an important role: positive effects of caloric restriction on the memory of rodents and humans have been demonstrated in intervention trials (see review by Zainuddin and Thuret [168]) and intermittent fasting has been proposed as promising approach for promoting brain health and preventing neurodegenerative disease in a recent review [179]. Intermittent fasting could exert beneficial effects by reducing insulin resistance, improving metabolic regulation, increasing autophagy, reducing inflammation and neuroinflammation, and increasing BDNF [179,180].

## 8. Systemic Inflammation, Nutrients and Neuroinflammation

Systemic immune activation appears to contribute significantly to the pathogenesis of AD outside the brain [17,100]: increased serum concentrations of inflammation markers like neopterin, sTNF-R75 and sIL2-R have been documented in patients with AD [181,182,183]. Neopterin is produced by macrophages upon stimulation with the proinflammatory cytokine interferon gamma (IFN-γ) in the elderly and to a greater extent in AD [184,185]. Neopterin was also documented to be elevated in patients with mild cognitive impairment [186], suggesting that systemic immune activation is already enhanced in the beginning of AD. 

**Figure 3:**
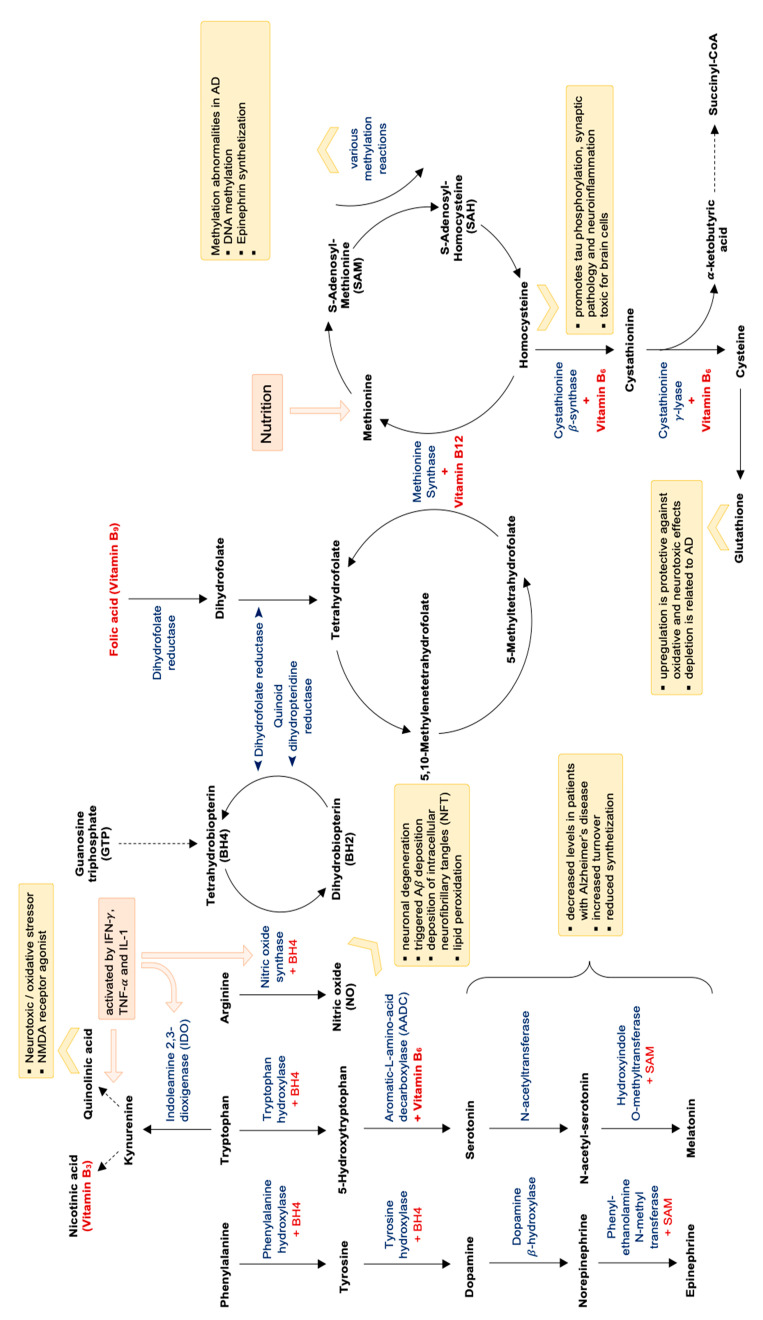
Methylation cycle, folate cycle, oxidative stress and neurotransmitter synthetization and dependent on vitamin B levels. Abbreviations: NMDA = *N*-methyl-D-aspartate; AD = Alzheimer’s disease; SAM = S-Adenosyl-Methionine; BH4 = Tetrahydrobiopterin; CoA = coenzyme A.

In parallel, the T-helper cell type 1 (Th1) cytokine IFN-γ also activates the enzyme indoleamine 2,3-dioxygenase-1 (IDO-1), which catalyzes tryptophan breakdown via the kynurenine pathway [182,184,187]. Tryptophan degradation was shown to be accelerated in AD [182]. One study also demonstrated that immune-mediated tryptophan breakdown, neopterin formation and lipid peroxidation coincide in patients with AD and are correlated with each other [188]. Enhanced lipid peroxidation is mostly due to oxidative stress, which is an important consequence of chronic immune activation [182]. Neopterin has been proposed to be a good marker for oxidative stress, and in fact high neopterin concentrations were shown to be associated negatively with concentrations of B-vitamins folic acid and vitamin B12, which are oxidized easily and irreversibly [182]. Furthermore, Tetrahydrobiopterin, which is an important cofactor for the formation of catecholamines and for nitric oxide metabolism, is easily oxidized by free radicals, suggesting that alterations of the dopamine and adrenaline axis as well as decreased vessel reactivity are linked with oxidative stress (see also Figure 3). 

Decreased availability of B vitamins folic acid, vitamin B12, vitamin B3 and also vitamin B6—probably also produced insufficiently by intestinal microbiota [189]—impairs the function of many important biochemical pathways: As B vitamins are essential cofactors for the formation of neurotransmitters serotonin, but also melatonin and vitamin B3 (niacin), conversion of the substrates to the end products can be impaired by low B vitamin availability (see Figure 3). Also the production of catecholamines like, e.g., adrenalin is dependent on a sufficient supply of vitamin B6 (for the conversion of tyrosine to L-DOPA) as well as vitamin C, magnesium, iron and the pteridine Tetrahydrobiopterin (THB). 

If folate and vitamins B6 and B12 are diminished, also concentrations of homocysteine rise [190]. Homocysteine is toxic for brain cells, several studies in animals could show that homocysteine can enhance neurodegeneration [191]. Already earlier elevated homocysteine concentrations have been shown to coincide with low B vitamin levels and elevated concentrations of Th1 type immune activation marker neopterin [192] in the blood of AD patients. Interestingly, supplementation of obese patients with delayed-type nicotinic acid (vitamin B3) was shown to improve biomarkers for inflammation and systemic insulin sensitivity going along with an increase in the abundance of *Bacteroidetes* [193]: both inflammation and insulin resistance have been demonstrated to contribute importantly to neurodegeneration in AD [194].

An international consensus statement classified elevated plasma total homocysteine concentrations as modifiable risk factor for the development of cognitive decline, dementia, and Alzheimer’s disease in older persons [190]. Several prospective studies reviewed in this consensus statement could demonstrate a gradient relationship between increasing homocysteine levels and cognitive impairment or dementia [195,196,197,198]. Although the question, whether homocysteine per se is the risk factor for cognitive decline or whether it is just a marker for deficiency of the vitamins folic acid, vitamin B6 and B12 [199] remains to be answered, measurement of homocysteine concentrations appears as cheap and simple method to determine a significant risk factor for cognitive decline. Treatment with B vitamins is not only effective to lower homocysteine, but also to slow down the rate of whole and regional brain atrophy and of cognitive decline markedly (as reviewed by Smith et al. [190]). Therefore, the consensus statement also advised to treat moderately raised plasma total homocysteine (>11 μmol/L) in elderly with B vitamins, which were regarded as easy, inexpensive, and safe to treat therapy option.

However, not only decreased B vitamin availability might impair human metabolism, but also enhanced immune-mediated tryptophan degradation and the depletion of other important nutrients: An adequate tryptophan supply e.g., is essential for protein biosynthesis and also to down-regulate immune activation cascades [200]. In patients with cardiovascular disease elevated neopterin concentrations were associated with lower tryptophan concentrations [201], higher homocysteine concentrations and reduced B vitamin concentrations [202] and a worse survival [203,204]. Elevated neopterin concentrations also were demonstrated to coincide with decreased concentrations of vitamin C, E [205] and vitamin D [206] indicating that in patients with cardiovascular disease chronic immune activation/inflammation goes along with decreased vitamin concentrations, which are probably due to an increased consumption of vitamins. 

Similarly, also in patients with AD nutrient and vitamin deficiency is well documented: In a meta-analysis de Wilde and coworkers [207] describe a lower blood and CSF/brain availability of docosahexaenoic acid (DHA), choline, vitamin B12, folate, vitamin C and vitamin E. Furthermore, vitamin D concentrations are often low in AD patients and have been linked with the risk of cognitive decline and dementia in older adults [208,209]. As the brain is critically dependent on nutrient supply, a multi-component nutritional intervention was suggested [207]. A recent review suggested that beside patients’ nutritional state altered circulating amino acid levels in AD patients might also be due to Aβ deposition itself [210]. These alterations might lead to difficulties in muscle energy formation with increased aspartic acid consumption, muscle hypercatabolism and increased consumption of antioxidant systems [210].

## 9. Other Important Risk Factors for the Development of AD: Physical Activity, Sleep Disturbances, Chronic Stress and Environmental Toxins

However, recent studies show that also lifestyle appears to play an underestimated role in the development of AD and may also attenuate genetic risk factors: a recent study investigating the role of a healthy lifestyle and of genetic risk in nearly 200,000 patients without cognitive impairment at the age of 60 years could show that both, an unfavorable lifestyle and high genetic risk were linked with a higher risk to get dementia [211]. A favorable lifestyle (which was defined as no smoking, regular physical activity of at least 75 vigorous or 150 minutes moderate activity/week, a diet following recommendations on dietary priorities for cardiometabolic health and little alcohol consumption), on the other hand, was associated with a lower dementia risk—also in patients with high genetic risk [211]. The effects of different lifestyle factors and also of environmental toxins on cognition and microbiota are reviewed in the following subsections.

### 9.1. Physical Activity

A review by Stephen and co-workers [212] investigated the relationship between physical activity and incident AD: very interestingly, physical activity was inversely associated with the risk of AD in 18 studies, and leisure-time physical activity was superior to work-related physical activity. Already earlier, moderate but extended physical activity (aerobic fitness training) was shown to increase brain volume in aging humans [213], whereas low physical activity at midlife and also in the elderly was associated with a higher risk of dementia and AD [214]. Furthermore, results of a recently published prospective Korean study with more than 247,149 individuals followed up for 6 years confirm that continued regular physical activity in patients with minor cognitive impairment is associated with a protective effect against developing dementia/AD [215], mediated by greater gut microbiota diversity due to active lifestyle [216].

### 9.2. Sleep Disturbances

Furthermore, sleep disturbances appear to contribute importantly to the development of AD: a meta-analysis by Bubu and co-workers [217] revealed that individuals with sleep problems had a 1.68 times higher risk for the combined outcome of cognitive impairment and/or AD than those without sleep disturbances. In fact, approximately 15% of cases of AD in this population were even attributed to sleep problems [217]—which is alarming, as today many patients report about sleep problems. Sleep deprivation is very critical for the health due to several reasons: it does not only change circadian rhythms, but also goes along with an increased activity of the sympathetic nervous system and hypothalamic-pituitary-adrenal axis (HPA axis), exerts important metabolic effects (e.g., it increases the risk for obesity [218]) and also induces pro-inflammatory responses [219]. Short-term sleep disruption goes along with an increased stress responsitivity, somatic pain, reduced quality of life, emotional distress and mood disorders, and cognitive, memory, and performance deficits in otherwise healthy adults [219]. Experiments show that sleep deprivation (relative to baseline) results in a significant increase in Aβ burden in the right hippocampus and thalamus and that these increases are associated with mood worsening [220]. 

Poor regeneration during sleep can impair learning and memory significantly, as it decreases neuronal plasticity and also neurogenesis [221]. It has been shown that functional neurons can be generated from adult neural precursors throughout life in the hippocampus and other regions of the brain [222]. The hippocampus appears to be especially vulnerable/sensitive to chronically restricted and disrupted sleep [223]. Thus, impaired hippocampal plasticity and reduced hippocampal cell proliferation (neurogenesis) may contribute importantly to cognitive disorders and psychiatric diseases [221,224]. During sleep, hormones like insulin-like growth factor 1 (IGF-1), growth hormone (GH), melatonin as well as BDNF, are upregulated and can promote hippocampal neurogenesis [225]. Cortisol, which in high concentrations inhibits neurogenesis in the hippocampus, is downregulated during normal sleep. This fact may also explain why prolonged sleep deprivation—as caused by shift working—superinduces the sympathetic system and impairs cognitive function [225]. Additionally, the composition, diversity and metabolic function of the gut microbiota are significantly altered in patients with insomnia compared to healthy individuals [226]. Increased sleep efficiency and total sleep time are positively correlated with microbiome diversity, whereas waking after sleep onset was negatively correlated with diversity [227]. Sleep efficiency, IL-6 concentrations and abstract thinking were better in patients with high numbers of *Bacteroidetes* and *Firmicutes*, while other taxa (*Lachnospiraceae, Corynebacterium, and Blautia*) were negatively correlated with sleep measures [227]. 

### 9.3. Chronic Stress

Furthermore, persistent over-activation of the HPA axis by chronic stress appears to be involved crucially in cognitive decline [228], possibly also by modulating the microbiota: chronic stress was shown to decrease the number of *Lactobacilli* [229]. 

An early dysregulation of the HPA-axis—closely interacting with the gut microbiota—has been documented well in AD patients and leads to an over-secretion of cortisol. Glucocorticoids easily enter the brain and negatively affect the hippocampus and also the prefrontal cortex, thereby supporting cognitive decline occurring in AD [228]. Chronic stress is critically involved in the development and progression of neurodegenerative disease [230]: via dysregulation of the HPA axis, stress can accelerate extracellular Aβ plaque deposition and intracellular tau hyperphosphorylation [231,232,233] in mouse models for AD. Additionally, it can also trigger co-morbid depression in neurodegenerative diseases [234,235]. Chronically elevated corticosteroid levels were demonstrated to alter the morphology of neurons and reduce hippocampal volume in rats [236,237]. Increased cortisol levels are found in patients with early-stage AD and have been related with a more rapid progression of AD [238]. High cortisol plasma levels were even identified as reliable biomarker for AD [239]. Higher baseline CSF cortisol levels in AD patients were associated with faster clinical worsening and cognitive decline [240]. High cortisol levels consecutive to acute high intensity stress were furthermore shown to come along with impaired working memory and visio-spatial memory [241]. HPA dysfunction has also been related with an increased intestinal permeability, motility and mucus production [242], which would indicate that also gut microbiota might be affected. In fact, data of animal models also indicate that microbiota alterations are involved: In mice exposed to chronic psychosocial stress disruptions in neurologic function were associated with complex shifts in the microbiota and altered immunoregulatory responses [243]. Chronic stress decreased the overall diversity of the microbiome with significantly lower levels of *Coriobacteriaceae* and tendentially lower levels of *Akkermansia muciniphilia* [243].

Interestingly, administration of *lactobacillus helveticus NS8* improved chronic restraint stress-induced behavioral (anxiety and depression) and cognitive dysfunction in a rat model, showing a similar (or even better) effect as citalopram [244]. Additionally, treatment with *lactobacillus helveticus NS8* also went along with lower plasma corticosterone and adrenocorticotropic hormone levels, higher plasma interleukin-10 levels, restored hippocampal serotonin and norepinephrine (NE) levels, and more hippocampal BDNF mRNA expression than in chronic stress rats. [244].

Persistent over-activation of the HPA axis also impairs adult hippocampal neurogenesis and shrinkage. Therefore, AD has been proposed to be a progressive disease leading to irreversible damage of the hippocampus, and later on also brain areas in the cortex [245]. While earlier studies proposed that neurogenesis is only possible during the embryonal and perinatal phase, it is now well established that functional neurons can be generated from adult neural precursors throughout life in the hippocampus and other regions of the brain [222]. Thus, aging alone does not appear to be causal for the development of AD [246], but rather facilitates it by enforcing processes, that finally lead to neuroinflammation and neurodegeneration (see review by Nehls [246]). 

In fact, animal and human studies could demonstrate, that diverse stressors can negatively affect the composition of microbiota [247,248,249], with, e.g., an increase of *Clostridiales* and a decrease of *Lactobacilli* [250]. Gubert and co-workers have reviewed the effects of stress, diet and exercise as modulators of gut microbiota recently very comprehensively and conclude that “environmental modulation” of gut microbiota should be investigated by further studies, as it may provide novel therapeutic approaches for neurodegenerative and psychiatric disorders [251]. 

### 9.4. Environmental Toxins

Environmental toxins may in fact represent another, so far completely underestimated problem afflicting and troubling our gut microbiota and also our brain. Smoking, which has long been recognized as dangerous due to the inhalation of various toxins, can alter gut microbiota profiles by increasing *Bacteroides-Prevotella* species [252]. However, also effects of other environmental toxins on gut microbiota and on mental health should be investigated in more detail: recent reviews depict that neurotoxic metals such as lead, mercury, aluminum, cadmium and arsenic, as well as some pesticides and metal-based nanoparticles (NP) are involved in AD pathogenesis, as they are able to increase Aβ peptide and the phosphorylation of Tau protein (P-Tau), causing amyloid plaques and neurofibrillary tangles characteristic of AD [253,254]. 

A recent review describes altered gut microbiota composition and metabolic profiles as consequence of heavy metal exposure, and also states that perturbations of the gut microbiota by heavy metals may cause metabolic diseases [255]. Gut microbiota can affect the absorption and metabolism of heavy metals, but chronic exposure induces gut dysbiosis, which can be counter-acted by probiotics [255].

In mice and avian experiments chronic glyphosate exposure affected the gut microbiota negatively, especially at a younger age [256,257]. Chronic glyphosate treatment induced an increase of anxiety and depression-like behaviors and altered the gut microbiota composition in terms of relative abundance and phylogenic diversity significantly in mice: *Corynebacteria, Firmicutes, Bacteroidetes and Lactobacilli* were diminished in treated mice [256]. Pre- and neonatal exposure to glyphosate, which also has antibiotic properties, impaired the fertility of mice and resulted in changes of maternal behavior in exposed mothers [256]. However, the effects on offspring were even more alarming: glyphosate suppressed potentially beneficial microbes at an early age and glyphosate exposed animals showed a consecutive decrease of locomotor activity, sociability, learning and short- and long-term memory as adult mice—as well as alterations of their cholinergic and dopaminergic systems [256]. Activated microglia and astrocytes were seen in glyphosate exposed offspring, and neuroinflammation was demonstrated in the medial prefrontal cortex and hippocampus going along with cognitive abnormalities [256]. A recent review discusses effects of various different environmental pollutants and molecular mechanisms involved in the pathogenesis of neurological disorders [258].

Already earlier it was recognized that bacteria-dependent metabolism of pollutants modulates the toxicity of environmental toxins for the host [259], as gut microbes have a high capacity to metabolize environmental chemicals. On the other hand, environmental contaminants like silver nanoparticles used in the food industry are able to alter the composition and/or the metabolic activity of the gastrointestinal bacteria strongly [259]. 

To summarize, there is an increasing number of “environmental stressors”, which can endanger the integrity of our human/microbiota ecosystem. In fact, it appears crucial to realize that our lifestyle also significantly influences our gut microbiota: dysbiosis of gut microbiota can induce systemic immune response and neuroinflammation, but also alter metabolism importantly (e.g., by inducing insulin resistance). These alterations can be compensated by the body for many years, but during aging capacities to compensate wane and irreversible damage occurs—finally going along with progressive cognitive decline.

## 10. Summary and Future Perspectives

AD has been regarded as a disease which only derives from the CNS without influence of the periphery for many decades. The core pathophysiological mechanisms are still described as accumulation of the abnormal proteins Aß and hyperphoshorylated tau [3]. However, more recent reports suggest an important role of peripheral infections by oral and intestinal bacteria that induce systemic inflammatory cascades which then lead to neurological damage by eliciting neuroinflammation [260]. Microbiota control the basic aspects of immunity and behavior in health and disease [261]: a well-balanced and diverse gut microbiota is necessary for normal stress responsivity, sociability, and cognition. Furthermore, it maintains CNS homeostasis by regulating intestinal permeability, immune function and BBB integrity. Neurotransmitter synthesis, synaptic, and neurotrophic signaling systems and neurogenesis are crucially influenced by gut microbiota. 

The dysbiotic intestinal microbiome produces a multi-component mixture of microbial metabolic products, which significantly activate the immune system and increase the production of cytokines and inflammatory mediators [41]. These compounds decrease the integrity of the intestinal mucosa barrier and the BBB, intensify systemic inflammatory reactions, and induce amyloid aggregation. Decreased tightness of the intestinal mucosa and increased BBB permeability by the “wrong composition” of gut microbiota or a reduced diversity in the elderly thereby facilitate the entry of a large amount of bacterial amyloid and LPS into circulation and CNS, where inflammatory cascades induce neuropathological disorders with an amyloidogenic component [41].

Moreover, it is likely that infections like CMV may be a trigger for neuro-inflammatory processes in the brain [262]. Chronic peripheral infections and dysbiotic intestinal microflora may lead to many pathological changes in different tissues, also distant from the source of infection [9,103]. Initially, the body is able to resist to these alterations. However, during aging and mediated by different unhealthy lifestyle factors (like an unhealthy diet, e.g., standard WD, decreased physical activity, chronic stress, sleep disturbances or environmental toxins) the regenerative capacity and ability to restrict infections decrease, leading to neurodegenerative processes and finally to dementia. Thus, pathological changes may start in and outside the brain many years before clinical symptoms of AD appear. 

Determining the host-microbiota interactions in more detail will enable advances in personalized medicine and in developing probiotics fitting individualized patterns [263,264,265]. Future strategies will hopefully create disease-modifying drugs as well as probiotics with prebiotics and dietary supplements [266,267] in the presymptomatic stages of AD before development of overt dementia. Possible treatment options will be reviewed in the following chapter.

## 11. Therapeutic Options/Perspectives to Slow Down Cognitive Decline

So far, treatment options for AD primarily have concentrated on single drugs that interfere with glutamatergic and cholinergic neurotransmission [3]. However, these drugs only address single biochemical pathways at a late stage of disease. Recent data showing the influence of gut microbiota indicate that new treatment strategies in patients with mild cognitive impairment might be more useful to slow down or even reverse cognitive decline: the gut provides the largest physical interface between the environment (including the microbiome) and the individual. More detailed clinical investigations of the microbiota–gut–brain communication and of the role of specific probiotic bacterial strains are necessary. Identification of gastrointestinal microorganisms using metagenomic and metabolomic methods can increase the ability to examine more subtle interactions between host and microbiome [268]. Further animal studies will enable us to better assess the role of different nutrients, but also of environmental toxins/stressors on the gut microbiota, the immune and also the neuro-endocrine system: these data will allow us to judge effects on the “human–microbiota ecosystem”, which are urgently needed. Furthermore, effects of dietary interventions like vitamins/micronutrients as well as secondary plant compounds and probiotics on the microbiota and inflammation status should be investigated in more detail. 

Recent studies are also beginning to address this interesting and promising topic and will be reviewed in the following. Last, but not least, multi-domain intervention approaches addressing different lifestyle (and genetic) factors will be described and personalized medicine as interesting new treatment option will be proposed.

### 11.1. Vitamins and Micronutrients

Nutrient deficiency is a well-established problem in AD [207]. Supplementation of patients with deficient substances (ideally targeting documented deficiencies by prior laboratory diagnosis) appears reasonable. As already mentioned above, B vitamins are recommended as easy, inexpensive, and safe to treat therapy to treat moderately raised plasma total homocysteine (>11 μmol/L) in elderly, especially in patients with cognitive deficits [269]. B-vitamins are not only essential for the proper functioning of many biochemical pathways in the host, but also for the viability/metabolism of certain auxotrophic bacterial strains of the gut microbiota: B-vitamin sharing has been proposed to promote the stability of gut microbial communities [270]. However, there is little information so far about possible effects of B vitamin supplementation on the microbiota so far. To our knowledge only data investigating effects of delayed-release nicotinic acid (Vitamin B3) in obese patients are available: supplementation with delayed-release nicotinic acid was able to increase the abundance of *Bacteroidetes* and improve biomarkers for systemic insulin sensitivity and metabolic inflammation [193]. 

A recent systematic review of randomized controlled trials investigating whether other dietary interventions had effects on the cognition of AD patients concluded that omega-3 fatty acid showed positive effects at different doses [271]. This might be partly related to effects of the gut microbiome since omega-3 fatty acids were shown to influence the abundance of *Faecalibacteria, Bacteriodetes* and *Lachnospiraceae*. It is suggested that the interplay between gut microbiota and omega-3 fatty acids helps to maintain the integrity of the intestinal wall and exerts beneficial effects on the interaction with host immune cells [272]. Furthermore, an oral supplement with a combination of eicosapentaenoic acid (EPA), docosahexaenoic acid (DHA), phospholipids, uridine monophosphate, choline, selenium, and vitamins B6, B12, B9, C, and E seemed to be effective in the early stages of AD. 

According to a 2019 Cochrane review there is also moderate quality evidence from a single study that vitamin E supplementation may slow down functional decline in AD [273]. A very interesting 10-week pilot study investigated the effects of a broad spectrum micronutrient administration on the fecal microbiome content in young patients with attention-deficit/hyperactivity disorder [274]: the differential abundance and relative frequency of *Actinobacteria* significantly decreased post-micronutrient treatment, and the diversity of the microbiome increased [274].

### 11.2. Plant-Derived Substances and Polyphenols

Ginseng, inositol and specialized nutritional formulas seem to have a positive effect on cognition [271]. Ginseng was actually shown to enhance the growth of beneficial microbiota including *Lactobacillus* and *Bacteroides* [275]. Additionally, inositol was shown to increase microbial SCFA and butyrate production and enhance the growth of *Lactobacilli* [276]. Although the quality of these interventions was low to moderate, more randomized and controlled studies with secondary plant ingredients like dietary polyphenols might be interesting, especially with polyphenols and curcumin. These pluripotent substances are mostly potent radical scavengers, modulate pro-inflammatory cascades (as reviewed by Dias et al. [277] and Monje et al. [151]) and can even influence amyloid formation [278,279]. In fact, a double-blind placebo-controlled pilot study in non-demented humans could demonstrate that a bioavailable form of curcumin led to significant memory and attention benefits [280]. Furthermore FDDNP-PET scans performed before and after 18 months of curcumin treatment suggested that behavioral and cognitive benefits were associated with decreases in plaque and tangle accumulation in brain regions modulating mood and memory [280]. These promising results appear worth being tested in larger prospective trials [281]. 

Both resveratrol and curcumin modulate gut microbiota and could be useful for the treatment of metabolic syndrome [282,283] and associated conditions like AD in a combined therapeutic concept [284]. Curcumin treatment in humans considerably changes the ratio between beneficial and pathogenic bacteria by increasing the abundance of *Bifidobacteria, Lactobacilli,* and reducing the loads of pro-inflammatory *Enterobacteria* and *Enterococci* [283]. Curcumin exerts diverse neuroprotective effects (see review by Cole et al. [285]) via different pathways, e.g., by reducing amyloid and inhibiting amyloid toxicity [285]. Additionally, curcumin is very effective to enhance the synthesis of another essential brain nutrient, namely DHA [286].

Similarly, resveratrol has potent antioxidant, anti-inflammatory, anticarcinogenic, anti-aging and neuroprotective effects (which were reviewed recently by Arbo et al. [287]), but in clinical trials so far effects on cognition were not significant—possibly due to the low bioavailability of resveratrol [287]. Still, a higher dietary intake of flavonoids has been associated with a reduced risk of developing Alzheimer dementia [288,289,290]. Flavonoids could further change the composition of gut microbiota by increasing the growth of *Bifidobacteria* and *Lactobacilli* [291], especially by fermentation procedures of fruit or vegetables rich in antioxidant plant compounds, e.g., fermented papaya was shown to reduce oxidative stress in AD patients, thereby probably reducing neuronal damage [292]. Furthermore, mango was shown to positively affect intestinal microbiota by increasing the presence of *Faecalibacterium*, *Bifidobacterium*, *Prevotella* and *Bacteriodes* which are beneficial for human gut health [293]. A recent study further demonstrated that pomegranate stimulated the growth of *Akkermansia munciniphila,* which plays an important role in the breakdown of phenolic intestinal compounds and may thereby contribute to its prebiotic effects [294]. Positive effects of various single nutrients on AD are summarized in Table 1—combination supplements are discussed above. 

### 11.3. Probiotics

The first study (double-blind, placebo-controlled) performed in patients with AD showed that supplementation with *Lactobacillus acidophilus, Lactobacillus casei, Bifidobacterium bifidum*, and *Lactobacillus fermentum* resulted in a significant improvement in MMSE score [304]. In addition, decreases in plasma concentrations of serum high-sensitivity C-reactive protein, malondialdehyde, insulin resistance, and serum triglycerides were encountered compared to the control group [304]. Another Iranian study examining probiotic and selenium co-supplementation showed a significant increase in MMSE score in patients with AD, in parallel also a significant reduction in hsCRP, lower insulin levels and a significant increase in total antioxidant capacity and glutathione levels was seen [305]. 

Very recent animal studies [306,307], clinical trials [308] and a meta-analysis of randomized controlled clinical trials [309] also suggest that altering microbiota with probiotics can lead to changes in brain function including cognitive functions. Mice in an early stage of AD were treated with a probiotic formulation, that decreased their cognitive decline compared with controls and reduced accumulation of amyloid β aggregates [310]. Furthermore, the probiotics affected the composition of gut microbiota, plasma concentrations of inflammatory cytokines and key metabolic hormones [310]. Furthermore, results of a recent Austrian study suggest that probiotics have beneficial effects on cognition and mood [311]: healthy students taking a multi-strain probiotic during a stressful period of exams performed better in recognition tasks than students who got placebo or without treatment (85% recognition compared to 70%). Furthermore, they were happier and functional brain MRI scans showed that brain regions for memory, motor function and attention were better perfused in students taking probiotics than in those with placebo or no treatment [311]. In emotional decision-making processes participants from the probiotic group showed increased alertness and were more confident and definite in making decisions. Interestingly, microbiome composition was related with scores for response accuracy, depressiveness and hopelessness. 

In an earlier exploratory intervention study, supplementation of a multispecies probiotic for 4 weeks resulted in a significant increase of the anti-inflammatory fecal bacterial strain *Faecalibacterium prausnitzii* in patients with AD. In parallel zonulin, a leaky gut marker significantly decreased by this intervention [90], while inflammatory markers (neopterin, Kynurenine/tryptophan ratio) slightly increased.

Previous animal studies showed that administration of probiotics induced changes in genes involved in inflammation and neural plasticity with a positive impact on neural functions [312]. Mice treated with probiotics showed higher plasma levels of gut hormones ghrelin and leptin: ghrelin counteracts memory deficits and synaptic degeneration in AD animal models [313], and leptin acts as neurotrophic factor and exerts neuroprotective effects against toxicity induced by Aβ oligomers in vitro [314]. Oral intake of probiotics furthermore also showed beneficial consequences on mood and psychological distress in rats and healthy human volunteers [315]. As depression and chronic stress are frequently observed in patients with AD, and supplementation with *Lactobacillus helveticus NS8* was effective to improve chronic stress-induced depression/anxiety and cognitive dysfunction also in a mouse model [244], probiotic supplementation might be beneficial to treat several symptoms of AD patients in parallel. 

Still, despite these promising data, further and larger clinical trials are necessary to assess the efficacy of probiotics in AD patients more exactly. A study performed in patients with severe AD could not observe significant changes regarding cognition, inflammation markers (TNF-a, IL-6) and oxidative stress markers (malondiadehyde, glutathione, total antioxidant capacity) after 12 weeks of probiotic supplementation [316]. Probably, effects of probiotic supplementation depend on several variables: the composition of the probiotic supplement, the number of bacteria used, and of course also the severity of dementia or other co-morbidities of patients driving inflammation. Additionally, also the diet of patients, supplementation with vitamins, micronutrients, phytochemicals, and physical activity, as well as the load of environmental toxins and stress might influence the effects of probiotic supplementation. 

Fecal microbiota transplantation (FMT) is increasingly applied as a therapeutic in conditions associated with gut dysbiosis. FMT of fecal microflora from healthy individuals to patients with AD could help restore intestinal microflora and reduce the negative impact of the dysbiotic microbiome on the gut and brain function, though human FMT studies in neurodegenerative diseases are still lacking [41,317,318]. 

However, as FMT is an invasive method, other less invasive methods should be tried first: in fact, dietary intervention strategies like adherence to a MD appear to be very effective to improve cognitive decline and enlarge the diversity of our gut microbiota (see also chapter 7).

### 11.4. Multi-Domain Intervention and Personalized Medicine

To modify unfavorable lifestyle factors, multi-domain intervention with changes of diet, increased physical activity, cognitive training and the monitoring of vascular risk factors appears as very promising therapeutic option: in the large, long-term, randomized controlled FINGER trial this therapeutic approach was effective to improve cognitive functioning in at-risk elderly people from the general population compared to a control group (general health advice) [319]. This multi-domain lifestyle intervention shall now also be tested by the The World-Wide FINGERS (WW-FINGERS) global network for dementia risk reduction and prevention, in over 25 countries [320]. 

However, as adherence decreases with increasing intervention complexity and intensity [321], personalized medicine approaches might be the treatment of choice: in fact, the neurologist Dale Bredesen, who is involved in AD research since many decades, has developed such a personalized treatment approach. He proposes that AD (like other chronic illnesses) is an age—associated network imbalance featuring many underlying mechanisms, and many or all of these mechanisms may need to be addressed therapeutically for optimal clinical efficacy [322]. Therefore, he has developed a novel, comprehensive, and personalized therapeutic program, which addresses underlying metabolic alterations and also overwhelming inflammatory cascades [323,324]. He suggests to first diagnose metabolic alterations, nutrient deficiencies and inflammation by various laboratory analyses/functional brain scans/cognitive tests. Results of these laboratory analyses and tests show, which processes (local and systemic inflammation, wrong diet [leading to dysybiosis, systemic inflammation, deficiency of essential nutrients, insulin resistance, neuroinflammation], chronic stress, sleep disturbances, toxins and hormonal imbalances) are dysregulated [322]. In response to these underlying causes patients are recommended to, e.g., modify their diet, do more sports, reduce stress by yoga or take supplements or hormones for diagnosed deficiencies. Additionally, also the adequate treatment of infections (e.g., of periodontitis) and the elimination of environmental toxins are scheduled in this program. This personalized treatment ensures that patients are treated with necessary and effective measures, so that they also adhere to this multi-domain therapy.

This “system biological approach” by Bredesen has already been shown to be effective in pilot studies: in a pilot study with 10 patients cognitive decline could be reversed in nine patients (Bredesen 2014). Furthermore, a follow-up study with results of 100 treated patients (treated by different physicians) could demonstrate that the concept works [325]. To our mind, this treatment approach for AD appears to be very promising and should be investigated in further clinical trials enrolling more patients. However, not only Bredesen, but also other scientists signify the importance of personalized medicine in AD patients. Peng and coworkers suggest that in-depth information (from genome sequencing, brain imaging, peripheral biomarkers, and even functional assays on neurons derived from patient-specific induced pluripotent cells) in combination with demographic information (APOE genotype, age, gender, education, environmental exposure, life style, and medical history) will enable a better understanding of the disease mechanisms underlying AD and facilitate the design of personalized stage specific therapeutic intervention in AD [326]. 

Times are changing and in the “age of chronic diseases” system biological treatment approaches using personalized medicine therapy should be examined for their efficacy by well conducted clinical trials to guarantee sustainable and effective medical care.

## Figures and Tables

**Table 1:** Positive effects of various nutrients on AD.

Nutrient	Positive Effect	Reference
omega-3 fatty acid	smaller decline in MMSE and ADAS-cog	Freund-Levi et al. [295] Quinn et al. [296] Shinto et al. [297]
ginseng	improvement in MMSE and ADAS-cog	Lee et al. [298] Heo et al. [299]
inositol	improvement in orientation and language	Barak et al. [300]
curcumin	improvement in MMSE and MWM	Voulgaropoulou et al. [281] Mishra et al. [301]
flavonoids	decreased incidence of AD within 20 years of FU	Holland et al. [290]
vitamin B_6_	better performance of verbal memory	An et al. [302]
folate acid	lower risk of AD development better cognitive reserve for global cognition, verbal memory and attention	Lefèvre-Arbogast et al. [303] An et al. [302]
vitamin E	slower functional decline after 48 months of FU	Farina et al. [273]

MMSE = Mini-Mental State Exam; ADAS-cog = Alzheimer’s Disease Assessment Scale-Cognitive Subscale; MWM = Morris Water Maze test; AD = Alzheimer’s disease; FU = Follow-up.
